# BC094916 suppressed SP 2/0 xenograft tumor by down-regulating Creb1 and Bcl2 transcription

**DOI:** 10.1186/s12935-018-0635-7

**Published:** 2018-09-14

**Authors:** Ruonan Xu, Ying Fang, Chunmei Hou, Bing Zhai, Zhenyu Jiang, Ning Ma, Liang Wang, Gencheng Han, Renxi Wang

**Affiliations:** 1 College of Life Science and Technology, Xinjiang University, Urumqi, 830046 Xinjiang China; 2 Laboratory of Immunology, Institute of Basic Medical Sciences, P.O. Box 130 (3), Taiping Road #27, Beijing, 100850 China; 3 Department of Rheumatology, First hospital of Jilin University, Changchun, 130021 China; 4 Department of Geriatric Hematology, Chinese PLA General Hospital, Beijing, 100853 China

**Keywords:** BC094916, B cells, Plasma cells, Multiple myeloma, Autoimmune diseases, SLE

## Abstract

**Background:**

Both multiple myeloma (MM) and systemic lupus erythematosus (SLE) are associated with abnormal production of plasma cells, although their pathological mechanism of each disease is different. The main characteristic of both diseases is uncontrolled differentiation of B cells into plasmablast/plasma cells. Despite continuous research on prognostic factors and the introduction of new agents for MM and SLE, treatments still do not exist for controlling plasmablast/plasma cells. Thus, it is necessary to identify novel therapeutic targets of plasmablast/plasma cells. Because of its plasmablast-like characteristics, the mus musculus myeloma SP 2/0 cell line was used in this study to test the effect of a novel therapeutic agent (BC094916 overexpression) on plasmablast/plasma cells.

**Methods:**

We first determined gene expression profiles of plasma cells using Affymetrix microarrays and RNA-sequencing. The effect of BC094916 on SP 2/0 cell proliferation, cell cycle, and apoptosis was determined by CCK8 and fluorescence-activated cell sorting. The SP 2/0 xenograft mouse model was used to assess the impact of BC094916 on tumor progression. The luciferase reporter system was used to evaluate the effect of BC094916 on Creb1 and Bcl2 transcription.

**Results:**

We found that BC094916 mRNA was decreased in plasma cells. The mouse myeloma cell line SP 2/0 expressed low levels of BC094916 mRNA, whereas BC094916 overexpression suppressed SP 2/0 cell proliferation by inducing apoptosis. BC094916 overexpression suppressed tumor progression in the SP 2/0 xenograft mouse model. We also found that BC094916 mediate apoptosis by suppressing transcription of the Creb1 and Bcl2 genes, which promote the transcription of eukaryotic translation initiation and elongation factor genes.

**Conclusions:**

BC094916 overexpression suppressed Creb1 and Bcl2 transcription to induce cell apoptosis, which suppressed SP 2/0 proliferation and xenograft tumor progression. Thus, BC094916 overexpression may be a potential therapeutic agent for treatment of MM and autoimmune diseases such as SLE.

## Background

B cells play an important role in the adaptive immune system. Upon migration to secondary lymphoid organs (i.e., spleen or lymph nodes), B cells that encounter antigen either differentiate into short-lived plasma cells or enter a germinal center (GC) where they further differentiate into high-affinity antibody-producing plasma cells and memory B cells [1–3]. DNA-binding transcription factors, including PAX5, BCL6, Blimp1, and Xbp1, are involved in B cell differentiation and plasma cell fates [4]. Correct regulation of B cell activity and function is essential for humoral immunity. If regulatory mechanisms are disrupted, B cells can contribute to the development of some autoimmune diseases such as SLE and B cell malignancies. The mechanisms that control B-cell differentiation are subject to transcriptional regulation, but, overall, are not well-defined, which leaves a gap in our understanding of the development of B-cell-related diseases [5].

The discovery of B-cell involvement in diseases such as SLE, multiple sclerosis (MS), rheumatoid arthritis (RA), and type 1 diabetes represents a paradigm shift in our understanding of the pathogenesis of these diseases, which still lack mechanistic explanations [6]. Therapies that target B cellsas appear to be effective for treating autoimmune diseases. Rituximab-mediated B-cell depletion has been used successfully to treat autoimmune diseases, an outcome that promoted the development of additional B cell targeting agents [7]. B cell–activating factor (BAFF) is involved in the development of autoimmune disorders [8]. In 2011, the Food and Drug Administration approved belimumab, an anti-BAFF monoclonal antibody (mAb), for treatment of SLE. We and other researchers demonstrated that an additional BAFF inhibitor, atacicept (TACI-IgG), promotes B-cell depletion [9–11]. BAFF-specific targeted therapy affects early-stage B cells in the periphery without affecting late-stage compartments, such as memory or bone marrow plasma cells [12]. Depletion of plasma cells has potential for treating autoimmune diseases [7].

MM is a neoplastic proliferation of plasma cells characterized by the presence of monoclonal immunoglobulins in blood and/or urine. Despite continuous research on the development of prognostic factors and new treatment agents MM remains an incurable and debilitating disease [13]. Even with recent advances in therapy regimens, MM patients commonly develop drug resistance and relapse. Monotherapy with PD-1 inhibitors produced disappointing results [14]. Due to the heterogeneous genetics of MM, it is likely that combinatorial treatment strategies will be needed, such as a combination of monoclonal antibodies, immune checkpoint inhibitors, vaccines, and chimeric antigen receptor (CAR) T-cell therapy [15]. Development of optimal combinatorial therapeutic strategies requires additional research into the identification of novel therapeutic targets.

Pathogenic plasma cells play a critical role in some autoimmune diseases and MM. To explore plasma cells as a novel therapeutic target, we determined the differences in gene expression between plasma cells and B cells and found that BC094916 mRNA was decreased in plasma cells. The BC094916 gene belongs to the Ifi200 cluster (Ifi202b, Ifi203, Ifi204, Ifi205, Mnda, Mndal, Aim2, Pydc4, Pyhin1, Pydc3, EG240921, LOC623121, AI607873, and EG666028) and contains the N-terminal DAPIN/PYRIN motif and/or a HIN200 domain that are characteristic of the Ifi family [16]. Proteins of the mammalian PYHIN family (IFI200/HIN-200) provide defense against infectious disease agents by recognizing foreign DNA [17]. BC094916 was shown to be associated with viral infection [18]. In addition, inhibitors of the renin–angiotensin–aldosterone system reduced BC094916 expression in dystrophic skeletal muscles [19]. However, the role of BC094916 in the viral infection or dystrophic skeletal muscles is unclear.

In this work, overexpression of BC094916 in the SP 2/0 xenograft mouse model induced apoptosis, which suppressed tumor progression. Mechanistically, BC094916 could function as a suppressor of transcription to reduce the expression of proteins such as Bcl2. Thus, overexpression of BC094916 may be a potential therapy for treating MM and autoimmune diseases such as SLE.

## Materials and methods

### Ethics committee approval

Care, use, and treatment of mice in this study were in strict agreement with international guidelines for the care and use of laboratory animals. This study was approved by the Animal Ethics Committee of Beijing Institute of Basic Medical Sciences.

### Mice

Seven-to-nine-week-old female or male C57BL/6 and Balb/c mice were purchased from the Chinese Academy of Medical Sciences (Beijing, China). Seven-to-nine-week-old female lupus-prone MRL/MpJ/lpr/lpr (MRL/lpr) mice were purchased from Nanjing Biomedical Research Institute of Nanjing University (Nanjing, China) and described previously [20]. EAE induction on C57BL/6 mice was performed, as previously described [10]. All mice were bred in our animal facilities under specific pathogen-free conditions.

### Treatment of lupus-prone mice with TACI-IgG

Treatment of lupus-prone mice with TACI-IgG was previously described [10]. Simply, Lupus-prone MRL/lpr mice were divided into the following two groups: 1, TACI-IgG treated; 2, control IgG-treated. Six lupus-prone mice per group were injected i.p. with 5 mg/kg TACI-IgG and control IgG (Rongchang pharmaceuticals, LTD, Shandong province, China) at 1, 2, 3, and 4 week (twice per week) after the mice reached 6 month of age.

### Cell sorting

The splenocytes were separated from three 7–9-week female C57BL/6 mice per group, stained with anti-mouse CD5 (clone no. 53-7.3) and B220 (clone no. RA3-6B2, eBioscience, USA) antibodies. CD5^+^ and CD5^−^ B220^+^B cells were sorted by flow cytometry (FACS). All flow cytometry data were acquired with FACSCanto, FACSCantoII, or FACSAria (BD Biosciences), gated on live lymphocyte-sized cells on the basis of forward and side scatter, and analyzed using FlowJo software (Tree Star, Ashland, OR). CD19^+^ and B220^+^ B cells were separated by CD19 (Cat No. 130-052-201) and B220 (Cat No. 130-049-501, Miltenyi Biotec, Germany) Microbeads, respectively.

### Cell culture and transfection

Mouse myeloma cell line SP 2/0 cells and human embryonic kidney HEK 293T cells were from the American Type Culture Collection (ATCC; Rockville, MD, usa) and described previously [21]. All cells were maintained in complete RPMI 1640 medium in a humidified 5% CO_2_ atmosphere at 37 °C. For cell transfection, BC094916 cDNA (General Biosystems, Anhui, China) was cloned into lentiviral vector LV201 or LV122 (Fugene Corp., Guangzhou, China) to generate BC094916 and BC094916-EGFP fusion protein, respectively. BC094916-expressing LV201 or LV122 was then transfected into SP 2/0 cells, and stable transfectants were identified by drug selection (Puromycin, Sigma, 10 μg/ml).

### B cells were stimulated in vitro with LPS

B cells were cultured in RPMI 1640 medium containing 10% FBS, 2 mM glutamine, penicillin (100 IU/ml), streptomycin (100 μg/ml), and 50 mM 2-mercaptoethanol. Cells were stimulated with 10 μg/ml LPS (Sigma L2630 from Escherichia coli 0111:B4; Sigma, St Louis, MO).

### Affymetrix microarrays

Affymetrix microarrays were performed by GMINIX Ltd (Shanghai, China) and described previously [22]. Total RNA was extracted from B cells with Trizol and purified over Qiagen RNeasy columns (Qiagen). Synthesis and labeling of RNA and hybridization of arrays were conducted. Stained arrays (430 2.0) were scanned on an Agilent Gene Array Scanner (Affymetrix).

### RNA sequencing

RNA was isolated from cells with Qiagen RNeasy Micro or Mini Kits (on the basis of cell number), according to the manufacturer’s instructions. RNA-seq was done with an Illumina HiSeq2500 instrument at Genewiz corp., Suzhou, China.

### Immunofluorescence and confocal microscopy

Cells were observed under a fluorescence microscope or seeded onto glass coverslips in 24-well plates, washed with PBS, fixed in 4% formaldehyde solution and permeabilized with 0.2% Triton X-100/PBS. Cells were blocked with 2% BSA in PBS for 30 min. Coverslips were incubated with DAPI and observed under a confocal laser scanning microscope.

### Measure of cell proliferation with cell counting kit-8 (CCK8) assay

Measure of cell proliferation with cell counting kit-8 (CCK8) assay was described previously [23]. CCK8 kit was purchased from Dojindo Molecular Technologies, Inc. Rockcille, MD, USA. Briefly, 100 μl of cell suspension (5000 cells/well) in a 96-well plate were cultured for an appropriate length of time (e.g., 0, 1 or 2 days) in a humidified incubator (e.g., at 37 °C, 5% CO_2_). 10 μl of CCK-8 solution was added to each well of the plate and the plate was incubated for 1–4 h in the incubator. Measure the absorbance at 450 nm using a microplate reader.

### Quantitative PCR analysis

Quantitative PCR analysis has been described in our previous studies [22, 24]. Briefly, total RNA was extracted from B cells with Trizol (Invitrogen Life Technologies). The final RNA pellets were dissolved in 0.1 mM EDTA (2 μl/mg original wet weight). Reverse transcription reactions were carried out on 22 μl of sample using superscript II RNAse H-Reverse Transcriptase (Invitrogen Life Technologies) in a reaction volume of 40 μl. All samples were diluted in 160 μl nuclease-free water. qPCR was employed to quantify mouse gene expression from the cDNA samples. Mouse gene expression was normalized to the levels of the β-actin gene.

### Immunoblot analysis

Whole-cell lysates were prepared for Western blotting. Twenty-five micrograms of cell protein were electrophoretically separated on a 10% SDS–polyacrylamide gel and transferred to a PVDF membrane, which was then blocked by incubation for 1 h at room temperature in 5% fat-free dry milk in Tris-buffered saline containing 0.1% Tween-20 (TBS-T). The blots were then incubated overnight at 4 °C with rabbit antibodies against anti-mouse GAPDH (KM9002, SunGene Biotech), Xbp-1 (ab37152, Abcam), blimp1 (sc-25380, Santa Cruz Biotech), Bcl2 (ab59348, Abcam), Bcl6 (sc-858, Santa Cruz Biotech), Aid (sc-25620, Santa Cruz Biotech), Myc (66004-1-lg, Proteintech), p53 (#2524, Cell Signaling Tech), Mcl1 (ab32087, Abcam), EGFP (ab290, Abcam) antibodies diluted 1:1000 in TBS-T containing 5% bovine serum albumin, washed for 25 min with TBS-T, and incubated for 1 h at room temperature with horseradish peroxidase-conjugated secondary F(ab’)2 (Zymed Laboratories, San Francisco, CA) (1:20 000 in TBS-T containing 5% bovine serum albumin), then bound antibody was visualized using the ECL detection system (Amersham, Arlington Heights, IL).

### Propidium iodide (PI)/FACS (cell cycle) analysis

Cell cycle analysis was described previously [22]. Cells were collected and washed 1 time with 5–10 ml of 1× PBS. Cells were suspended in 500 μl 1× PBS containing + 0.1% Glucose (at 4 °C) and 5 ml of cold 70% EtOH (kept at −20 °C) was immediately added, mixed, and kept at 4 °C for 1 h. Cell were then spun down and washed once with 1 × PBS (10 ml). Without adding more PBS, cells were then spun again for 2 min so that the residual PBS could be removed and cells were then suspended in 300 μl 69 μM propidium iodide (Cat# 537059, Calbiochem, San Diego, CA) solution with 38 mM Na Citrate (Cat# C7254 Sigma, St. Louis, MO) and 20 μl of 10 mg/ml RNase (Cat# R4875, Calbiochem, San Diego, CA). Cells were mixed, incubated at 37 °C for 30–45 min and analyzed by FACS.

### Annexin V/PI staining

Apoptosis detection kit was purchased from Sungenebiotech, Tianjing, China. Cells were centrifuged at 335×*g* for 10 min and resuspended in 2 ml 1× phosphate buffered saline (PBS) (no calcium, no magnesium). Cells were centrifuged at 335×*g* for 10 min and resuspended in 1 ml 1X Annexin V binding buffer. A total of 5 μl APC-conjugated Annexin V (Cat No. AO2001-02, Sungenebiotech, Tianjing, China) was added and the tubes incubated in the dark for 15 min at room temperature. A total of 100 μl of 1x Annexin V binding buffer was added to each reaction tube (final volume: ~ 200 μl). PI (4 μl, Cat No. AO2002-H, Sungenebiotech, Tianjing, China) was diluted 1:10 in 1x Annexin V binding buffer and a final PI concentration of 2 μg/ml was added in each sample. Tubes were incubated in the dark for 15 min at room temperature. 1× Annexin V binding buffer (500 μ) was added to wash the cells. Then the samples were ready to be analyzed by flow cytometry (FACS).

### SP 2/0 xenograft mouse model

To evaluate tumor growth in mouse models, 200 μl of cell suspension from 5 × 10^6^ SP 2/0 expressing GFP (vector) or SP 2/0 cells expressing GFP and BC094916 (BC094916) were subcutaneously injected into the left and right sides of the back of each Balb/c mouse. Mice were sacrificed on day 8 after the injection. Tumor volumes were determined by measuring the major (L) and minor (W) diameters with an electronic caliper. The tumor volume was calculated according to the following formula: tumor volume = π/6 × L × W^2^.

### Creb1 and Bcl2 promoter reporting gene analysis

Promoter reporting gene analysis has been described in our previous studies [25]. The firefly luciferase reporter plasmid pEZX-PG04.1 (Fugene Corp., Guangzhou, China) with the 5′-flanking region from start codon upstream − 1510 ~ + 173 of mouse Creb1 gene or − 1323 ~ + 160 of mouse Bcl2 gene. 0.5 μg Lv201/BC094916, 0.5 μg firefly luciferase reporter plasmids pEZX-PG04.1/Creb1 promoter or Bcl2 promoter (General Biosystems, Anhui, China), and 0.05 μg Renilla luciferase reporter vector pRL-SV-40 vector (cat# E2231, Promega Corp.) were co-transduced into 4 × 10^5^ SP 2/0 or 293T cells in 12-well plate by using 6 μL Lipofectamine^®^2000 Reagent (Cat# 11668-019, Invitrogen Corp.). On day 3, sequential measurement of firefly luciferase (Reporter #1) followed by Renilla luciferase activity (Reporter #2) was assessed on 1420 Multilabel Counter (1420 Victor 3, PerkinElmer Corp.), and analyzed. The results were shown as the ratio of firefly to Renilla luciferase activity.

### Statistics

Statistics were analyzed by using GraphPad Prism (version 5.0, GraphPad Software Inc., USA). The data were shown as mean ± standard error of the mean (SEM). Student’s t test was employed to determine significance between two groups (paired or unpaired) and Two-Way ANOVA analysis was used to determine significance among several groups. Differences were considered statistically significant when p < 0.05.

## Results

### Decreased expression of BC094916 in plasma cells and SP 2/0 cells

Previous studies demonstrated that B-cell-depletion therapy does not affect B cells in the late-stages of differentiation (e.g., plasma cells) [10, 12]. To identify novel therapeutic targets of plasma cells, gene expression profiling experiments were performed with Affymetrix microarrays. In B220^+^ cells derived from atacicept (TACI-IgG)-treated lupus-prone MRL/lpr mice, expression of plasma cell-associated transcription factors including Prdm1 and Xbp1 was increased, whereas expression of GC B cell-associated transcription factor Bcl6 and the mature B cell-associated transcription factor Pax5 was decreased (Table 1). Additionally, expression levels of a novel gene BC094916 were reduced in a similar ratio to that of Bcl6 and Pax5 (Table 1). These results suggest that BC094916 expression may be decreased in plasma cells. To investigate this possibility, B cells were treated with LPS to stimulate plasmablasts/plasma cell maturation [26] And gene expression patterns of plasma cells were determined using Affymetrix microarrays. In plasma cells, expression of Prdm1 and Xbp1 were increased, whereas Bcl6 and Pax5 expression was decreased (Table 2). Additionally, expression of BC094916 was reduced similarly to Bcl6 and Pax5 (Table 2). These results suggest that expression of BC094916 was decreased in LPS-stimulated plasmablasts. To evaluate BC094916 expression in lymphoblasts, the lymphoblast-like mouse myeloma cell line SP 2/0 was used in gene expression studies. SP 2/0 cells expressed high levels of Prdm1 and Xbp1 and low levels of Bcl6 and Pax5 (Table 3). SP 2/0 cells expressed low levels of BC094916 mRNA (Table 3). These results suggest that MM cells including SP 2/0 cells, do not express BC094916. Next, BC094916 expression patterns were evaluated in naïve B cells. We found that BC094916 mRNA in naïve B cells was at an intermediate level, between CD19 and Pax5 (highest expression level) and Bcl6, Prdm1, and Xbp1 (lowest expression level) (Table 4). To evaluate BC094916 expression in activated B cells, activated B cells were sorted from CD5^+^ B cells (CD5 is a B cell activation antigen [27, 28]) and EAE mice (a model of multiple sclerosis (MS) mouse model in which B cells are activated with myelin components emulsified in appropriate adjuvants [29]). BC094916 expression was slightly increased in activated B cells from CD5^+^ B cells or EAE mice (Table 5). Collectively, these data demonstrated that BC094916 expression was slightly increased when B cells were activated. Once B cells differentiated into plasma cells, BC094916 was not expressed.

**Table 1:** BC094916 mRNA expression decreased in TACI-IgG-regulated plasma cells

Gene symbol	Gene description	Fold change	Regulation
Prdm1	PR domain containing 1, with ZNF domain	15.73	Up
Xbp1	X-box binding protein 1	4.88	Up
Pax5	paired box gene 5	0.19	Down
Bcl6	B cell leukemia/lymphoma 6	0.20	Down
BC094916	cDNA sequence BC094916	0.14	Down

Three lupus-prone mice per group were injected i.p. with 5 mg/kg TACI-IgG or control IgG at 1, 2, 3, and 4 weeks (two times per week) after mice reached 6 months of age. On day 4 after therapy, mice were euthanized and B cells were separated from the spleen by B220 microbeads. The transcripts in B cells were determined by Affymetrix Microarrays. The fold change of the transcripts in TACI-IgG treatment to those in IgG group is shown

**Table 2:** BC094916 mRNA expression decreased in LPS-induced plasma cells

Gene symbol	Fold change		Regulation
	Experiment 1	Experiment 2	
Prdm1	34.05	27.52	Up
Xbp1	10.17	8.79	Up
Pax5	0.09	0.29	Down
Bcl6	0.16	0.17	Down
BC094916	0.04	0.11	Down

The splenocytes were separated from three C57BL/6 mice per group. B cells were sorted by B220 microbeads, and stimulated for 3 days in vitro by 10 μg/ml LPS. The transcripts in LPS-stimulated B cells were determined by Affymetrix Microarrays. The fold change of the transcripts in LPS-stimulated group to unstimulated group is shown from 2 separate experiments

**Table 3:** BC094916 mRNA did not express in SP 2/0 cells

Gene symbol	Total exon fragments	FPKM
Prdm1	1720.99	27.05
Xbp1	4553.99	159.74
Pax5	35	0.31
Bcl6	116.99	2.36
BC094916	0	0

The transcripts in SP 2/0 cells were determined by RNA-sequencing. Total exon fragments and FPKM (FPKM = total exon fragments/mapped reads (millions)/exon length (KB), Fragments per Kilo bases per Million reads) values of selected genes encoding Prdm1, Xbp1, Pax5, Bcl6 and BC094916 are shown

**Table 4:** B cells expressed the high level of BC094916 mRNA

Gene symbol	Total exon fragments	FPKM
CD19	20,349	441.5
Pax5	30,458	255
Bcl6	1865	27.73
Prdm1	244	2.9
Xbp1	2008	54.8
BC094916	798	19.65

The splenocytes from three 7–9-week female C57BL/6 mice per group were sorted by CD19 microbeads. The transcripts in B cells were determined by RNA-sequencing. Total exon fragments and FPKM (FPKM = total exon fragments/mapped reads (millions)/exon length (KB), Fragments per Kilo bases per Million reads) values of selected genes encoding CD19, Pax5, Bcl6, Prdm1, Xbp1, and BC094916 are shown

**Table 5:** BC094916 mRNA expression slightly increased in activated B cells

Gene symbol	Fold change		Regulation
	CD5^+^B/CD5^−^B	EAE/control	
BC094916	2.21	1.49	Up

The splenocytes were separated from three 7–9-week female C57BL/6 mice per group and CD5^+^B cells were sorted by FACS (the second column). B cells were sorted by CD19 microbeads from the splenocytes of three 11-week female EAE and control mice (the third column). The transcripts in B cells were determined by Affymetrix Microarrays. The fold change of the transcripts in CD5^+^B cells to CD5^−^B cells or CD19^+^B cells from EAE mice to those from control mice is shown

### BC094916 overexpression induced apoptosis of SP 2/0 cells

**Fig. 1:**
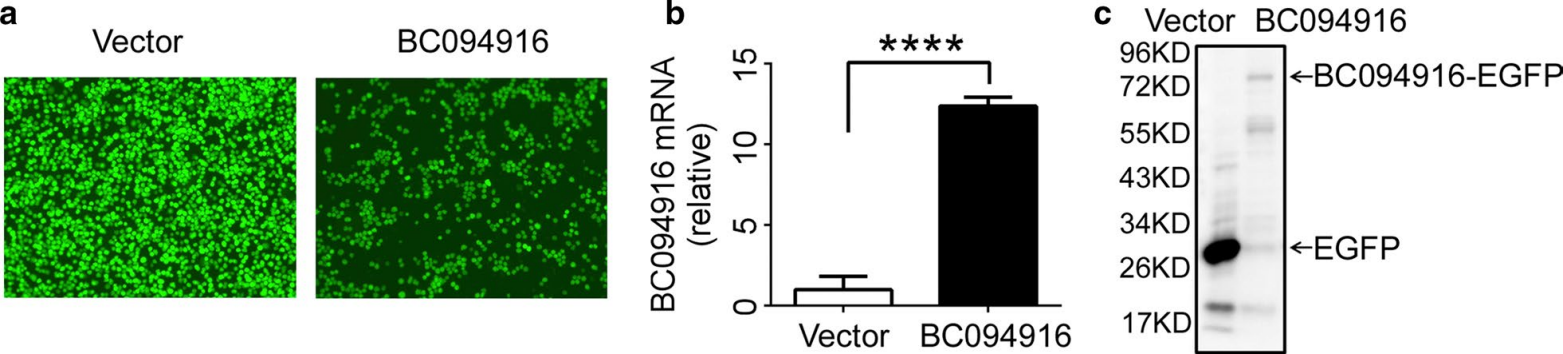
BC094916 overexpression in SP 2/0 cells. **a** Stable expression of BC094916 in SP 2/0 cells. BC094916- and EGFP-expressing lentiviral vector LV201 (BC094916) and only EGFP-expressing LV201 (vector) were transduced into SP 2/0 cells. Puromycin was used to select stable BC094916- and EGFP-expressing SP 2/0 cells. EGFP expression was shown. **b** BC094916 mRNA expression significantly increased in stable BC094916- and EGFP-expressing SP 2/0 cells. BC094916 mRNA expression was determined in the stable BC094916- and EGFP-expressing SP 2/0 cells (BC094916) and only EGFP-expressing SP 2/0 cells (vector) after cells were cultured for 48 h. **c** BC094916 protein expression significantly increased in BC094916-EGFP (fusion form of BC094916 carbon terminal and EGFP)-expressing SP 2/0 cells. BC094916-EGFP (BC094916)- or only EGFP (vector)-expressing lentiviral vector LV122 were transduced into SP 2/0 cells. At 72 h after transduction, cells were collected and anti-EGFP antibodies were used to probe EGFP or BC094916-EGFP fusion protein expression by western blot assay. **a**–**c** Data are representative of three independent experiments. **b** Two-tailed student’s t test. Error bars, SEM. ****p < 0.0001

To explore the therapeutic potential of BC094916 in plasma cell-associated diseases such as MM, BC094916 overexpressed in SP 2/0 cells. An LV201 construct expressing BC094916 and EGFP and a construct expressing only EGFP were transfected into SP 2/0 cells. Stable transfectants expressing BC094916 and EGFP, or EGFP alone were selected by puromycin (Fig. 1a). RNA-sequencing was used to determine expression profiles in SP 2/0 cells overexpressing BC094916. Relative to SP 2/0 cells expressing only EGFP, SP 2/0 cells expressing both BC094916 and EGFP overexpressed BC094916 mRNA (Table 6). This result confirmed that BC094916 was overexpressed in stably transfected SP 2/0 cells. To further confirm this, qPCR was used to analyze BC094916 mRNA expression. BC094916 mRNA was significantly increased in stably transfected SP 2/0 cells (Fig. 1b). To evaluate the BC094916 protein expression, a BC094916-EGFP fusion construct was generated in which EGFP was fused to the C-trminus of BC094916. Western blot analysis showed an approximate 80 kDa band corresponding to the BC094916-EGFP fusion protein (Fig. 1c). Collectively, these data demonstrated BC094916 overexpression in SP 2/0 cells.

**Table 6:** BC094916 overexpression suppress the expression of anti-apoptosis, eukaryotic translation initiation factor (Eif) and eukaryotic translation elongation factor (Eef) gene in SP 2/0 cells

Gene symbol	Total exon fragments			FPKM		
	Vector	BC094916	Fold change	Vector	BC094916	Fold change
BC094916	1.00	93.00	93.00	0.03	2.97	99.00
prdm1	1738.00	1280.00	0.74	19.86	16.59	0.84
Xbp1	6951.81	6944.75	1.00	173.37	197.54	1.14
Bcl6	223.71	125.99	0.56	3.37	2.16	0.64
Trp53	5333.00	4871.00	0.91	156.64	163.57	1.04
Bcl2	44.00	27.06	0.61	0.34	0.23	0.68
Bcl2l1	1499.00	954.00	0.64	37.95	29.11	0.77
Mcl1	35841.98	19278.00	0.54	850.34	493.80	0.58
Eif4a2	10798.10	7854.01	0.73	344.03	281.28	0.82
Eif5b	241.50	77.26	0.32	22.18	8.10	0.37
Eif4e2	989.06	507.78	0.51	42.48	24.37	0.57
Eif2s3x	307.22	0.00	0.00	14.79	0.00	0.00
Eef1b2	14673.00	9209.01	0.63	654.39	457.86	0.70
Eef1d	1175.02	582.81	0.50	90.55	51.18	0.57
Creb1	233.00	55.00	0.24	4.78	0.82	0.17

RNA-seq was done with an Illumina HiSeq 2500 instrument at GENEWIZ, Suzhou, China. Total exon fragments and FPKM (FPKM = total exon fragments/mapped reads (millions)/exon length (KB), Fragments per Kilo bases per Million reads) values of selected genes encoding prdm1, Xbp1, Pax5, Bcl6, Trp53, Bcl2l1, Bcl2l1, Mcl1, Eif4a2, Eif5b, Eif4e2, Eif2s3x, Eef1b2, Eef1d, and Creb1, and the fold change of gene expression in BC094916-overexpressed SP 2/0 cells to control SP 2/0 cells are shown

**Fig. 2:**
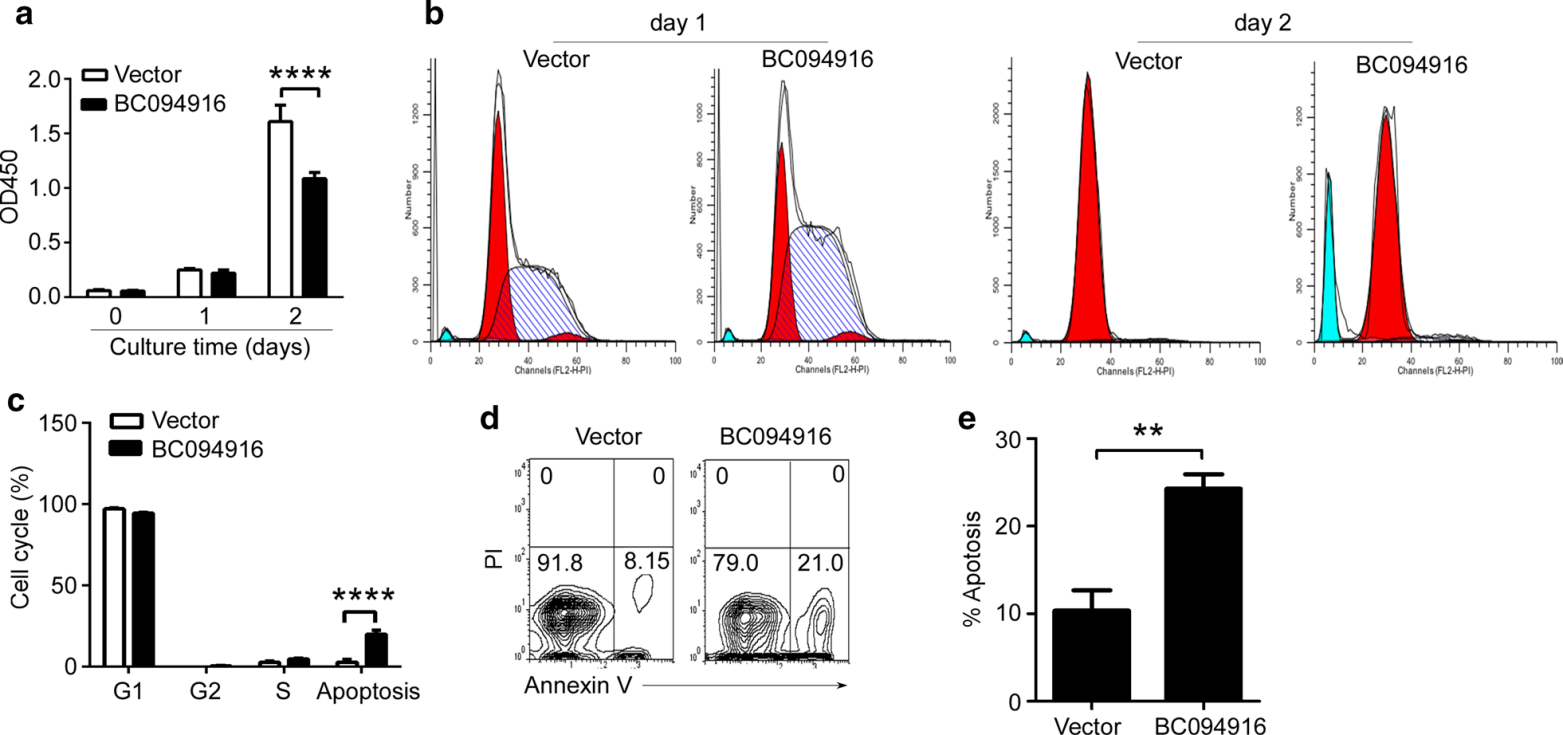
BC094916 overexpression reduced cell proliferation and promoted cell apoptosis. **a** BC094916 overexpression suppressed the proliferation of SP 2/0 cells. The stable BC094916- and EGFP-expressing SP 2/0 cells (BC094916) and only EGFP-expressing SP 2/0 cells (vector) were cultured in vitro for 0, 1, 2 days. CCK8 assay was used to determine the cell proliferation. **b**, **c** BC094916 overexpression did not affect the cell cycle of SP 2/0 cells. The stable BC094916- and EGFP-expressing SP 2/0 cells (BC094916) and only EGFP-expressing SP 2/0 cells (vector) were cultured in vitro for 1 and 2 days. Cells were collected, stained with propidium solution (PI) and analyzed by flow cytometry (FACS). FACS profile on day 1 and 2 (**b**) and the statistical analysis on day 3 (**c**) of cell cycle were shown. **d**, **e** BC094916 overexpression up-regulated the apoptosis in SP 2/0 cells. The stable BC094916- and EGFP-expressing SP 2/0 cells (BC094916) and only EGFP-expressing SP 2/0 cells (vector) were cultured in vitro for 2 days. Cells were collected, stained with Annexin V-APC and propidium solution (PI) and analyzed by flow cytometry (FACS). Representative FACS profiles (**d**) and the statistical analysis (**e**) of the percentages of apoptic cells were shown. **a**–**e** The data are representative of three independent experiments. **e** Two-tailed student’s t test and. **a**, **c** Two-Way ANOVA were followed by Bonferroni post-tests to compare each column to control column. Error bars, SEM. **p < 0.01, ****p < 0.0001

To explore the impact of BC094916 overexpression in SP 2/0 cells, the CCK8 assay was used to determine the role of BC094916 overexpression in cell proliferation. BC094916 overexpression significantly reduced SP 2/0 cell proliferation (Fig. 2a). To explore the mechanism by which BC094916 suppressed cell proliferation, cell cycle analysis was performed, which showed that BC094916 overexpression did not affect the cell cycle but induced cell apoptosis (Fig. 2b, c). To further evaluate the impact of BC094916 overexpression on apoptosis, Annexin V and PI staining and FACS were used to analyze apoptosis. The results suggest that BC094916 overexpression significantly promoted SP 2/0 apoptosis (Fig. 2d, e). Collectively, these data suggest that BC094916 overexpression significantly reduced SP 2/0 cell proliferation by inducing cell apoptosis.

### BC094916 overexpression suppressed tumor progression in the SP 2/0 xenograft mouse model

**Fig. 3:**
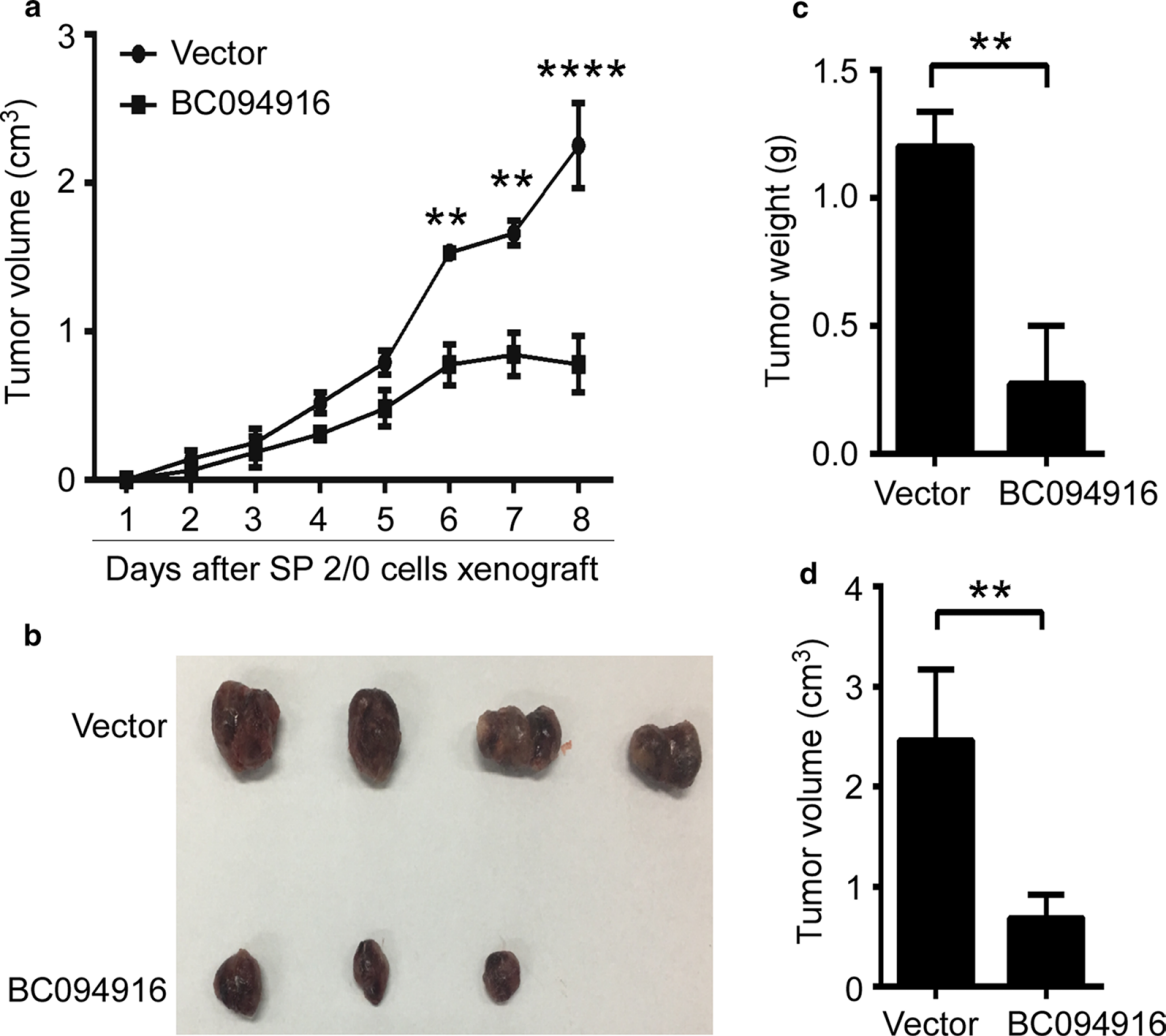
BC094916 overexpression suppressed tumor progression in the SP 2/0 xenograft mouse model. The stable BC094916- and EGFP-expressing SP 2/0 cells (BC094916) and only EGFP-expressing SP 2/0 cells (vector) were subcutaneously injected into Balb/C (4 mice/group) mice. **a** Tumor size from three independent experiments (n = 12 mice/group) was monitored every day by determining tumor volumes. **b**–**d** Pictures of subcutaneous tumor tissues **b** from one experiment (n = 4 mice/group), tumor weights (**c**) and tumor volumes (**d**) from three independent experiments (n = 12 mice/group) on day 8 after SP 2/0 xenograft. **a**, **c**, **d** Data are shown as mean ± SEM (n = 12) from three independent experiments. Two tailed Student’s t-test, **P < 0.01, ****p < 0.0001

To further determine the effect of BC094916 overexpression on in vivo tumor progression, the SP 2/0 xenograft mouse model was developed in Balb/c mice [21]. Stable SP 2/0 transfectants expressing both BC094916 and EGFP, or only EGFP were subcutaneously injected into Balb/C mice (5 × 10^6^ cells per mouse; 4 mice/group). Tumor volumes from each group were measured each day. BC094916 overexpression suppressed in vivo tumor growth and progression in Balb/c mice in a time-dependent fashion (Fig. 3a). Based on tumor size imaging, BC094916 overexpression reduced the size of SP 2/0 xenograft tumors in Balb/c mice (Fig. 3b). Tumor volumes and average tumor weights from each group were measured. The results showed that BC094916 overexpression reduced tumor volumes and weights in Balb/c mice (Fig. 3c, d). These data suggest that BC094916 overexpression suppressed SP 2/0 xenograft tumor progression.

### BC094916 is a transcriptional suppressor

**Fig. 4:**
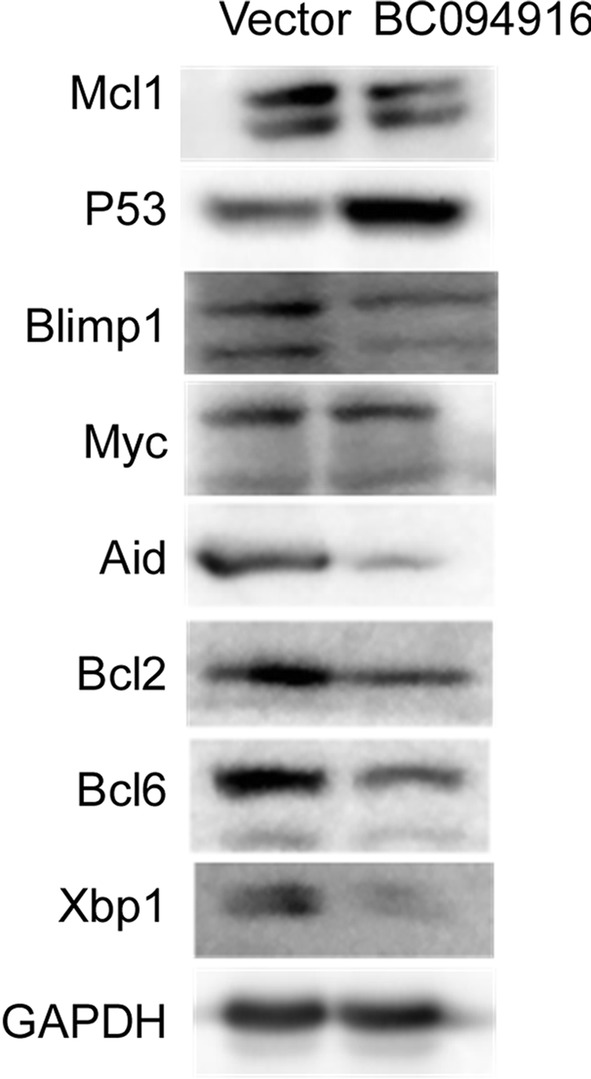
BC094916 overexpression suppressed anti-apoptosis proteins Bcl2 and Mcl1, plasma cells-related proteins Blimp1, Myc, Aid, Bcl6, and Xbp-1, and up-regulated apoptosis protein p53 in SP 2/0 cells. The stable BC094916- and EGFP-expressing SP 2/0 cells (BC094916) and only EGFP-expressing SP 2/0 cells (vector) were cultured for 2 days, collected and subject to immunoblot analysis with monoclonal anti-mouse Mcl1, p53, Blimp1, Myc, Aid, Bcl2, Bcl6, Xbp1 and GAPDH. Results represent three independent experiments

**Fig. 5:**
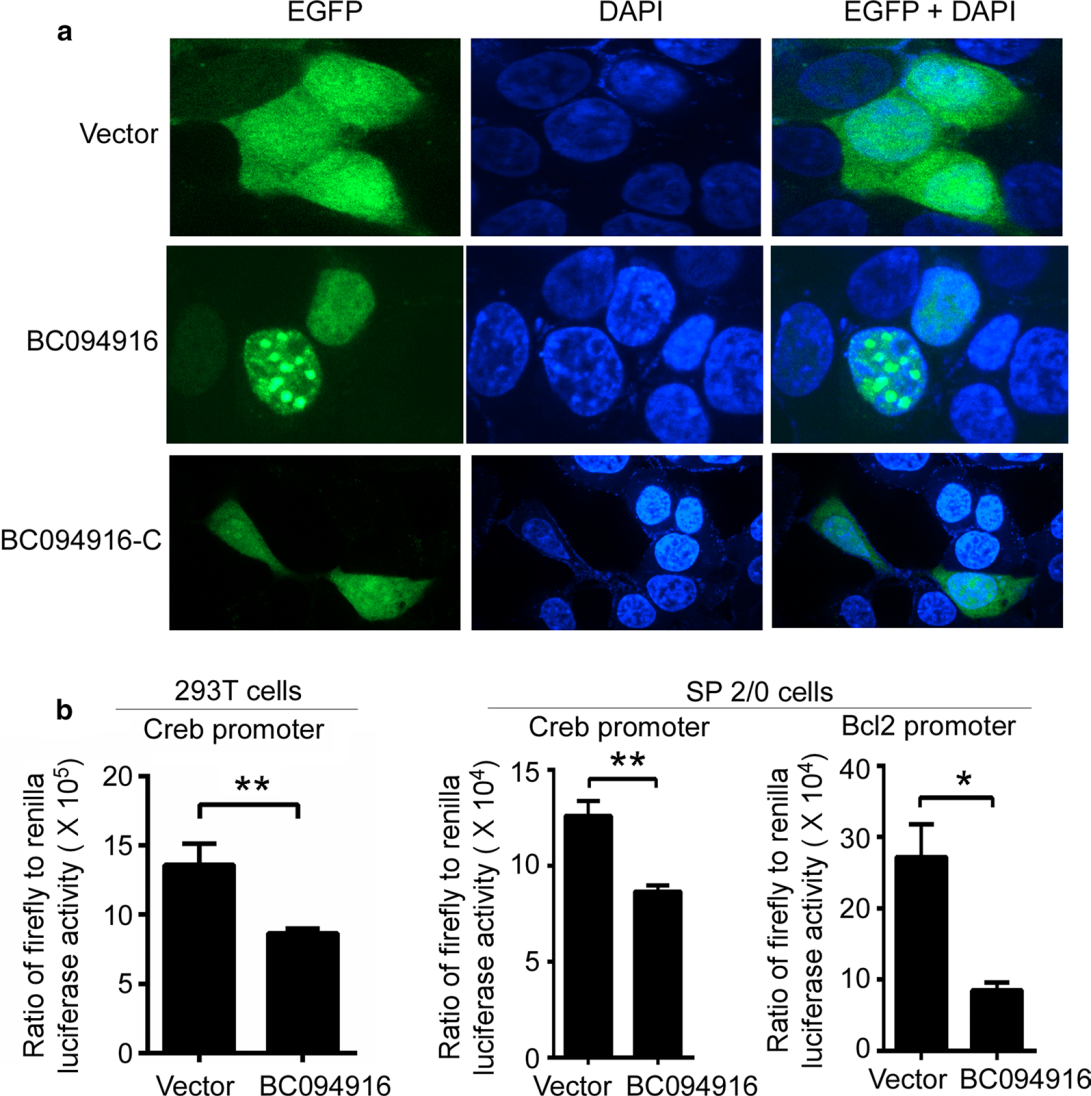
Transcription factor BC094916 suppressed the activation of Creb and Bcl2 promoter. **a** BC094916 located in the nuclear. 293T cells were transfected with BC094916-EGFP (BC094916)-, BC094916-C (the C-terminal of BC094916)-EGFP (BC094916-C) or only EGFP (vector)-expressing LV122, and cultured for 2 days. C ells were imaged and analyzed on a GE IN Cell Analyzer 2000. Representative images showed nuclear location of BC094916. **b** BC094916 suppressed the activation of Creb and Bcl2 promoter. BC094916 -expressing LV201 (BC094916) or empty vector LV 201 (Vector) and luciferase reporter vector pEZX-PG04.1/Creb1 promoter (− 1510 ~ + 173 bp) or pEZX-PG04.1/Bcl2 promoter (− 1323 ~ + 160 bp) were co-transduced into 293T (left panel) or SP 2/0 (middle and right panels) cells. Dual luciferase reporter gene expression was analyzed, and the results are shown as the ratio of firefly to Renilla luciferase activity. The data represent at least four independent experiments. Error bars, SEM. Two tailed Student’s t-test, *P < 0.05, **P < 0.01

The experiments described above suggest that BC094916 overexpression suppressed cell proliferation and SP 2/0 xenograft tumor progression by inducing cell apoptosis. To further explore the mechanisms underlying BC094916-induced apoptosis, western blot assays were performed to determine protein levels of the transcription factors Blimp1, Xbp-1, Myc, Aid, and Bcl6 duringdifferentiation of B cells into plasma cells, anti-apoptosis proteins Bcl2 and Mcl1, and apoptosis-induced p53 protein. BC094916 overexpression in SP 2/0 cells reduced levels of the anti-apoptosis proteins Bcl2 and Mcl1, plasma cell-related proteins Blimp1 and Xbp-1, and up-regulated the apoptosis protein p53 (Fig. 4). These results suggest that changes in expression of these proteins may be involved in cell apoptosis. RNA-sequencing was used to determine the effect of BC094916 overexpression on gene expression. mRNA levels of Blimp1, Xbp-1, and Trp53 were not changed (Table 6). BC094916 overexpression reduced expression of anti-apoptosis factors (Bcl2, Bcl1L1, Mcl1), eukaryotic translation initiation factors (Eif5b, Eif4e2, Eif2s3x), eukaryotic translation elongation factors (Eef1b2, Eef1d), and the transcription factor Creb1 in SP 2/0 cells (Table 6). These results suggest that BC094916 affected the transcription of some genes. To explore the mechanism by which BC094916 regulates transcription, we first determined the cellular location of BC094916. 293T cells were transfected with LV122 vectors expressing BC094916-EGFP (BC094916), BC094916-C (corresponding to C-terminal amino acids 262-393)-EGFP (BC094916-C), or only EGFP. Confocal images showed that EGFP and the C-terminus of BC094916 were distributed throughout the cells, whereas BC094916 localized to the nucleus (Fig. 5a). These results suggest that BC094916 may be a transcription factor. To further prove the possibility that BC094916 is a transcription factor, the role of BC094916 in the regulation of the Bcl2 and Creb1 promoters was evaluated. Activity of the Creb1 and Bcl2 promoters was analyzed using the luciferase reporter system in 293T and SP 2/0 cells (Fig. 5b). The results demonstrated that BC094916 could suppress activation of the Creb1 promoter in 293T and SP 2/0 cells (Fig. 5b, left and middle panels) and the Bcl2 promoter in SP 2/0 cells (Fig. 5b, right panels). These results indicate that BC094916 is capable of suppressing the Creb and Bcl2 promoters.

## Discussion

B cells expressed high levels of BC094916 mRNA (Table 4). When activated, B cells slightly up-regulated BC094916 expression (Table 5). When B cells differentiation into plasma cells/plastblasts, BC094916 expression was decreased (Tables 1, 2). These results are consistent with a previous study suggesting that follicle B cells expressed high levels of BC094916, whereas plastblasts expressed low levels of BC094916 [26]. Our results further indicated that SP 2/0 cells expressed low levels of BC094916 (Table 3). Futher studies should explore whether BC094916 expression is changed in autoimmune diseases and MM.

Targeted therapies, such as treatment with the BRAF inhibitor vemurafenib in BRAF-mutated MM patients or BCL-2 inhibition with venetoclax for MM with t [4, 14] are currently being used for treatment of relapsed and refractory MM (RRMM) [15]. Even with recent advances in therapy regimen, MM patients commonly develop drug resistance and relapse [30, 31]. To explore novel therapies that target plasma cells to treat autoimmune diseases and MM, BC094916 was overexpressed in SP 2/0 cells (Fig. 1). We found that BC094916 overexpression significantly suppressed SP 2/0 cell proliferation (Fig. 2) and SP 2/0 xenograft tumor progression (Fig. 3) by inducing cell apoptosis (Fig. 2). Three independent experiments (n = 4 mice/group/time, total n = 12 mice/group) of SP 2/0 xenograft tumor were performed. Collectively, we calculated and demonstrated an average from all three experiments (n = 12 mice/group) (Fig. 3a, c, d). Consistent with total results, BC094916 overexpression significantly suppressed tumor growth, tumor weights and tumor volume in each experiment (n = 4 mice/group) (data not shown). These results suggest that BC094916 overexpression may be an effective way to accomplish a gene-therapy treatment for MM. Gene therapy administered by transcatheter or percutaneous intratumoral injection is an emerging area of interest in tumor research. Some studies have entered the animal testing stage, including intratumoral gene therapy injections with a multipronged and multi-side hole needle for rectal carcinoma [32]. It is needed to explore and validated gene transfer systems to guide gene therapy and to evaluate the efficacy of promising therapies [15].

The human PYHIN proteins (AIM2, IFI16, IFIX, and MNDA) are critical regulators of immune responses, transcription, apoptosis, and the cell cycle [33]. Human IFI16, an ortholog of the mouse BC094916 gene, was shown to localize to the nucleus of 293T cells, lymphoma line Daudi cells, and IFNγ-treated cells of the leukemia line HL-60 [34]. In this work, BC094916 localized to the nucleus of 293T cells (Fig. 5a). Subcellular localization of IFI16 to the nucleus versus the cytoplasm (or both) depends on cell type. IFI16 protein can sense cytosolic as well as nuclear dsDNA and can initiate different innate immune responses (production of IFN-β versus proinflammatory cytokines) [34]. In this work, BC094916 was expressed in B cells (Table 4). In addition, BC094916 reduced Blimp1, Bcl6, Xbp1, Aid, Bcl2 and Mcl2 protein expression in SP 2/0 cells (Fig. 4). Thus, BC094916 may suppress differentiation of B cells into plasma cells. Thus, it is necessary to explore the role of IFI16 in differentiation of human B cells into plasma cells.

We found that BC094916 overexpression significantly reduced the transcription factor Creb1 in SP 2/0 cells (Table 6). In addition, BC094916 suppressed activation of the Creb gene promoter (Fig. 5b). A ChIP-qPCR assay demonstrated the presence of CREB binding sites in the EIF4A1 gene promoter, four binding sites in the promoter of EIF4E2, one CREB binding sites in the promoter of EEF1B2, and seven CREB binding site in the promoter of EEF1D (see http://saweb2.sabiosciences.com/chipqpcrsearch.php?facto). The Bcl-2 promoter region contains a CREB-binding site in its upstream promoter region [35, 36] and activation of CREB induces Bcl2 expression [37]. BC094916 overexpression suppressed expression of anti-apoptosis proteins (Bcl2, Bcl1L1, Mcl1), eukaryotic translation initiation factor (Eif5b, Eif4e2, Eif2s3x), eukaryotic translation elongation factors (Eef1b2, Eef1d) in SP 2/0 cells (Table 6). In addition, BC094916 also suppressed the activation of the Creb promoter (Fig. 5b). An ortholog of BC094916, human IFI16, inhibits HCMV replication by blocking the activity of Sp1-like transcription factors at the viral UL54 promoter [38, 39]. Collectively, these data demonstrated that BC094916 is a repressor of transcription.

Along with reduced expression of eukaryotic translation initiation and elongation factors by BC094916-mediated suppression of the Creb1 promoter, it is expected that Blimp1, Bcl6, Xbp1, Aid, Bcl2 and Mcl2 protein expression should also be reduced in BC094916-overexpressed SP 2/0 cells (Fig. 4). In addition, Bcl2 mRNA and protein levels were suppressed by BC094916. Several studies proposed that low levels of pro-apoptotic proteins determine apoptosis [40, 41]. Collectively, our data suggest that BC094916, a repressor of transcription, mediates cell apoptosis by suppressing transcription of Bcl2 and Creb1-regulated eukaryotic translation initiation and elongation factors.

## Conclusions

BC094916 mRNA was decreased in plasma cells and SP 2/0 cells, whereas BC094916 overexpression suppressed SP 2/0 cell proliferation and SP 2/0 xenograft tumor progression by inducing cell apoptosis. Mechanistically, BC094916 induced apoptosis by suppressing transcription of Bcl2, Creb1-associated eukaryotic translation initiation and elongation factors gene expression. Thus, BC094916 overexpression represents a novel therapy for MM and autoimmune diseases such as SLE.

## Abbreviations

SLE: systemic lupus erythematosus
MM: multiple myeloma
CCK8: cell counting kit-8
FACS: flow cytometry
GC: germinal center
MS: multiple sclerosis
RA: rheumatoid arthritis
BAFF: B cell-activating factor
LPS: lipopolysaccharide

## References

[1] Jacob J, Kelsoe G, Rajewsky K, Weiss U (1991). Intraclonal generation of antibody mutants in germinal centres. Nature.

[2] Vazquez MI, Catalan-Dibene J, Zlotnik A (2015). B cells responses and cytokine production are regulated by their immune microenvironment. Cytokine.

[3] Illera VA, Perandones CE, Stunz LL (1993). Mower DAJr, Ashman RF. Apoptosis in splenic B lymphocytes. Regulation by protein kinase C and IL-4. J Immunol..

[4] Nutt SL, Taubenheim N, Hasbold J, Corcoran LM, Hodgkin PD (2011). The genetic network controlling plasma cell differentiation. Semin Immunol.

[5] Scharer CD, Barwick BG, Guo M, Bally APR, Boss JM (2018). Plasma cell differentiation is controlled by multiple cell division-coupled epigenetic programs. Nat Commun..

[6] Fillatreau S (2018). B cells and their cytokine activities implications in human diseases. Clin Immunol..

[7] Hofmann K, Clauder AK, Manz RA (2018). Targeting B cells and plasma cells in autoimmune diseases. Front Immunol..

[8] Mackay F, Schneider P (2009). Cracking the BAFF code. Nat Rev Immunol.

[9] Nestorov I, Munafo A, Papasouliotis O, Visich J (2008). Pharmacokinetics and biological activity of atacicept in patients with rheumatoid arthritis. J Clin Pharmacol.

[10] Ma N, Xing C, Xiao H (2014). BAFF suppresses IL-15 expression in B Cells. J Immunol..

[11] Carbonatto M, Yu P, Bertolinom M (2008). Nonclinical safety, pharmacokinetics, and pharmacodynamics of atacicept. Toxicol Sci.

[12] Cancro MP, D’Cruz DP, Khamashta MA (2009). The role of B lymphocyte stimulator (BLyS) in systemic lupus erythematosus. J Clin Invest..

[13] Rajkumar S, Kumar S (2016). Multiple myeloma: diagnosis and treatment. Mayo Clin Proc.

[14] Tremblay-LeMay R, Rastgoo N, Chang H (2018). Modulating PD-L1 expression in multiple myeloma: an alternative strategy to target the PD-1/PD-L1 pathway. J Hematol Oncol..

[15] Nijhof IS, van de Donk NWCJ, Zweegman S, Lokhorst HM (2018). Current and new therapeutic strategies for relapsed and refractory multiple myeloma: an update. Drugs.

[16] Vogel H, Scherneck S, Kanzleiter T (2012). Loss of function of Ifi202b by a microdeletion on chromosome 1 of C57BL/6 J mice suppresses 11b-hydroxysteroid dehydrogenase type 1 expression and development of obesity. Hum Mol Genet.

[17] Cridland JA, Curley EZ, Wykes MN (2012). The mammalian PYHIN gene family: phylogeny, evolution and expression. BMC Evol Biol.

[18] Baranek T, Manh TV, Alexandre Y (2012). Differential responses of immune cells to type I interferon contribute to host resistance to viral infection. Cell Host Microb.

[19] Chadwick JA, Bhattacharya S, Lowe J, Weisleder N, Rafael-Fortney JA (2017). Renin-angiotensin-aldosterone system inhibitors improve membrane stability and change gene-expression profiles in dystrophic skeletal muscles. Am J Physiol Cell Physiol.

[20] Wang X, Wei Y, Xiao H (2016). Pre-existing CD19-independent GL7-Breg cells are expanded during inflammation and in mice with lupus-like disease. Mol Immunol.

[21] Liu X, Zhang Y, Wang Z (2016). Metabotropic glutamate receptor 3 is involved in B-cell-related tumor apoptosis. Int J Oncol.

[22] Ma N, Liu X, Xing C (2015). Ligation of metabotropic glutamate receptor 3 (Grm3) ameliorates lupus-like disease by reducing B cells. Clin Immunol..

[23] Zhu G, Liu X, Fang Y (2018). Increased mTOR cancels out the effect of reduced Xbp-1 on antibody secretion in IL-1a-deficient B cells. Cell Immunol.

[24] Wang X, Wei Y, Xiao H (2016). A novel IL-23p19/Ebi3 (IL-39) cytokine mediates inflammation in lupus-like mice. Eur J Immunol.

[25] Zhang Y, Wang Z, Xiao H (2017). Foxd3 suppresses IL-10 expression in B cells. Immunology.

[26] Shi W, Liao Y, Willis SN (2015). Transcriptional profiling of mouse B cell terminal differentiation defines a signature for antibody-secreting plasma cells. Nat Immunol.

[27] Miller RA, Gralow J (1984). The induction of Leu-1 antigen expression in human malignant and normal B cells by phorbol myristic acetate (PMA). J Immunol..

[28] Ying-zi C, Rabin E, Wortis HH (1991). Treatment of murine CD5-B cells with anti-Ig, but not LPS, induces surface CD5: two B-cell activation pathways. Int Immunol.

[29] Lalive PH, Molnarfi N, Benkhoucha M, Weber MS, Santiago-Raber ML (2011). Antibody response in MOG35-55 induced EAE. J Neuroimmunol.

[30] Yee AJ, Raje NS (2018). Panobinostat and multiple myeloma in 2018. Oncologist..

[31] Costello C, Mikhael JR (2018). Therapy sequencing strategies in multiple myeloma: who, what and why?. Future Oncol..

[32] Amalou H, Wood BJ (2013). Intratumoral gene therapy injections with a multipronged, multi-side hole needle for rectal carcinoma. Cardiovasc Intervent Radiol.

[33] Diner BA, Li T, Greco TM (2015). The functional interactome of PYHIN immune regulators reveals IFIX is a sensor of viral DNA. Mol Systems Biol..

[34] Veeranki S, Choubey D (2012). Interferon-inducible p200-family protein IFI16, an innate immune sensor for cytosolic and nuclear double-stranded DNA: regulation of subcellular localization. Mol Immunol.

[35] Wilson BE, Mochon E, Boxer LM (1996). Induction of bcl-2 expression by phosphorylated CREB proteins during B-cell activation and rescue from apoptosis. Mol Cell Biol.

[36] Pugazhenthi S, Miller E, Sable C (1999). Insulin-like growth factor-I induces bcl-2 promoter through the transcription factor cAMP-response element-binding protein. J Biol Chem.

[37] Shankar E, Krishnamurthy S, Paranandi R, Basu A (2010). PKCepsilon induces Bcl-2 by activating CREB. Int J Oncol.

[38] Dell’Oste V, Gatti D, Giorgio AG, Gariglio M, Landolfo S, De Andrea M (2015). The interferon-inducible DNA-sensor protein IFI16: a key player in the antiviral response. New Microbiol.

[39] Cristea IM, Moorman NJ, Terhune SS (2010). Human Cytomegalovirus pUL83 stimulates activity of the viral immediate-early promoter through its interaction with the cellular IFI16 protein. J Virol.

[40] Oltvai ZN, Korsmeyer SJ (1994). Checkpoints of dueling dimers foil death wishes. Cell.

[41] Oltvai ZN, Milliman CL, Korsmeyer SJ (1993). Bcl-2 heterodimerizes in vivo with a conserved homolog, bax, that accelerates programmed cell death. Cell.

